# Highly Virulent *Escherichia coli* O26, Scotland

**DOI:** 10.3201/eid1709.110199

**Published:** 2011-09

**Authors:** Kevin G.J. Pollock, Sheetal Bhojani, T. James Beattie, Lesley Allison, Mary Hanson, Mary E. Locking, John M. Cowden

**Affiliations:** Author affiliations: Health Protection Scotland, Glasgow, Scotland (K.G.J. Pollock, M.E. Locking, J.M. Cowden);; Yorkhill Hospital, Glasgow (T.J. Beattie, S. Bhojani);; Scottish *E. coli* O157/VTEC Reference Laboratory, Edinburgh, Scotland (L. Allison, M. Hanson)

**Keywords:** bacteria, verotoxin-producing Escherichia coli, VTEC, clinical sequelae, foodborne infections, Escherichia coli serotype O26, bacteria, hemolytic uremic syndrome, HUS, Scotland, letter

**To the Editor**: Hemolytic uremic syndrome (HUS) is a rare disorder characterized by microangiopathic hemolytic anemia, microthrombi, and multiorgan injury. HUS is one of the commonest causes of acute renal failure in children worldwide and is most frequently precipitated by infection with verotoxin-producing *Escherichia coli* (VTEC) such as *E. coli* O157 (*1*). However, non-O157 VTEC serotypes have been increasingly found in the development of HUS (*2**–**4*).

Although previous surveillance of childhood HUS in Scotland identified *E. coli* O157 in >90% of cases, non-O157 serotypes have also been associated with HUS (*5*). In 2010, several particularly severe cases of HUS were reported to Health Protection Scotland by a consultant pediatric nephrologist. Subsequent tests identified the pathogen in these cases as *E. coli* O26. However, in a recent study of pediatric HUS cases in Europe, children infected with *E. coli* O26 did not exhibit different clinical signs and symptoms from patients infected with other VTEC serotypes (*6*). To establish whether the host pathophysiologic responses to *E. coli* O157 and *E. coli* O26 strains differed, we analyzed a cohort of children with HUS who were infected with these VTEC serotypes.

In Scotland, most patients with pediatric thrombotic microangiopathy are referred to a specialist pediatric hospital, which immediately reports cases of HUS to Health Protection Scotland as part of national surveillance. To test the hypothesis that *E. coli* O26 was more virulent than *E. coli* O157, we performed an age-matched, nested case–case study of HUS patients and compared host clinical markers, treatment, and outcomes from pediatric cases in 2010. Data collection has been described elsewhere (*5*). The statistical significances of associations between categorical variables were investigated by using χ^2^, Fisher exact, Mann-Whitney, or *t* tests. All analyses were performed by using SPSS version 11 (SPSS Inc., Chicago, IL, USA) with a significance level of 5%.

Although initial signs and symptoms were similar for both sets of cases, i.e., bloody diarrhea and abdominal pain, statistical analysis showed that children with O26-HUS were more likely to have neurologic complications and diabetes mellitus and require admission to the intensive care unit than O157-HUS patients (p = 0.02 for neurologic complications and diabetes and p = 0.04 for admission to an intensive treatment unit; Table).

**Table:** Characteristics of infection in children with *Escherichia coli* O157 versus *E. coli* O26, Scotland, 2010*

Variable	O157 HUS, n = 12	O26 HUS, n = 3	p value
Age, y	4.5 ± 1.0	3.7 ± 1.3	
Clinical signs and symptoms			
Diarrhea	12	3	
Bloody diarrhea	8 (67)	2 (67)	NS
Abdominal pain	8 (67)	3 (75)	NS
Fever	2 (17)	1 (33)	NS
Neurologic involvement	3 (25)	3 (100)	0.02
Diabetes	1 (12)	2 (67)	0.02
Cardiomyopathy	0	1 (33)	0.04
Anuria, d	7.7 ± 2.4	15.7 ± 1.3	0.04
Clinical parameters			
Leukocyte count × 10^9^/L	42.1 ± 14.4	25.9 ± 4.8	NS
C-reactive protein, mg/L	93.5 ± 28.4	151 ± 74	NS
Serum albumin, g/L	22.5 ± 1.3	22.3 ± 2.4	NS
Lactate dehydrogenase, IU/L	2,521 ± 362	1,991 ± 642	NS
Admission to ITU	3 (25)	3 (100)	0.04
Duration of hospitalization, d	13.2 ± 2.2	25 ± 3.8	0.03
Treatment			
Peritoneal dialysis, d	7.4 ± 1.9	10	NS
Hemodialysis, d	8.5 ± 1.5	8.3 ± 3.0	NS
Hemofiltration	0	3 (100)	0.001
Laparotomy	0	1 (33)	0.04
Postdischarge hypertension	0	2 (67)	0.01

*Values are mean ± SE or no. (%). Significance relates to difference between groups. HUS, hemolytic uremic syndrome; NS, not significant, ITU, intensive treatment unit.

All patients with HUS were oligoanuric, and the 2 groups did not differ with respect to this parameter. However, O26-HUS patients had significantly longer periods of anuria than O157-HUS patients (p = 0.04; Table) and were more likely to require treatment with hemofiltration than with peritoneal or hemodialysis (p = 0.001; Table). One patient with O26-HUS also experienced cardiomyopathy resulting in reduced left ventricular function.

Our study illustrates the potential for increased severity of *E. coli* O26 infection in children. In Scotland, HUS is more commonly associated with *E. coli* O157 infection, and the outcome for children infected with this pathogen is much better than that reported in other studies (*7**,**8*). In this study, the clinical severity and outcomes for the children with O26-HUS were worse than for children requiring treatment for O157-HUS. We investigated the prehospital management of *E. coli* O157 and O26 patients in this cohort and found no difference in pharmacologic intervention or duration of delay in admission to hospital.

In our cohort, *vtx_1_* and *vtx_2_* genes were detected in isolates from 2 of 3 patients. A diagnosis was made in the third patient by detection of *E. coli* O26 lipopolysaccharide–specific immunoglobulin M in serum; it was therefore not possible to confirm the presence of *vtx* genes. However, it is not unusual for VTEC to be undetectable in stool samples from patients with HUS, most likely because of intrahost bacteriophage lysis. Therefore, serodiagnosis of VTEC is considered a necessary adjunct to bacteriological confirmation of infection (*9*). A recent study suggests *E. coli* O26 exists as a highly dynamic group of organisms that can undergo verotoxin gene loss and be transferred during infection in humans, resulting in new pathogenic clones (*10*). Therefore, *vtx_2_* gene acquisition by *E. coli* O26 may have contributed to increased virulence.

Our study was limited by the small number of patients with pediatric O26-HUS. However, given the severity of the complications experienced by the children in this cohort, we believe it is necessary to communicate these findings promptly to the international community.

We suggest that infection with *E. coli* O26 in children can result in more severe and complicated forms of HUS than those caused by *E. coli* O157. In contrast to the findings of Gerber et al., we found that there was a significant difference in neurologic complications between the 2 groups (*2*). Epidemiologic investigations found that 2 of the 3 children lived on farms and may have acquired infection while playing near their homes (the other was acquired through foreign travel). Risk communication of VTEC infection to parents of young children who live in farming communities remains problematic, perhaps because of the perception that immunity has been acquired. Although this suggestion may be true for adults, children are likely to be immunologically naive. Salient public health messages on simple precautionary behavior need to be regularly reinforced because prevention of VTEC infection prevents HUS.
